# Genetic Divergence of an Avian Endemic on the Californian Channel Islands

**DOI:** 10.1371/journal.pone.0134471

**Published:** 2015-08-26

**Authors:** Amy G. Wilson, Yvonne Chan, Sabrina S. Taylor, Peter Arcese

**Affiliations:** 1 Department of Conservation and Forest Sciences, University of British Columbia, Vancouver, BC, Canada; 2 ‘Iolani School, 563 Kamoku St., Honolulu, Hawaii, United States of America; 3 School of Renewable Natural Resources, Louisiana State University, Baton Rouge, Louisiana, United States of America; University of California Santa Cruz, UNITED STATES

## Abstract

The Californian Channel Islands are near–shore islands with high levels of endemism, but extensive habitat loss has contributed to the decline or extinction of several endemic taxa. A key parameter for understanding patterns of endemism and demography in island populations is the magnitude of inter–island dispersal. This paper estimates the extent of migration and genetic differentiation in three extant and two extinct populations of Channel Island song sparrows (*Melospiza melodia graminea*). Inter–island differentiation was substantial (G''_ST_: 0.14–0.37), with San Miguel Island having the highest genetic divergence and lowest migration rates. Santa Rosa and Santa Cruz Island populations were less diverged with higher migration rates. Genetic signals of past population declines were detected in all of the extant populations. The Channel Island populations were significantly diverged from mainland populations of *M*. *m*. *heermanni* (G''_ST_: 0.30–0.64). Ten mtDNA haplotypes were recovered across the extant and extinct Channel Island population samples. Two of the ten haplotypes were shared between the Northern and Southern Channel Islands, with one of these haplotypes being detected on the Californian mainland. Our results suggest that there is little contemporary migration between islands, consistent with early explanations of avian biogeography in the Channel Islands, and that song sparrow populations on the northern Channel Islands are demographically independent.

## Introduction

**Fig 1:**
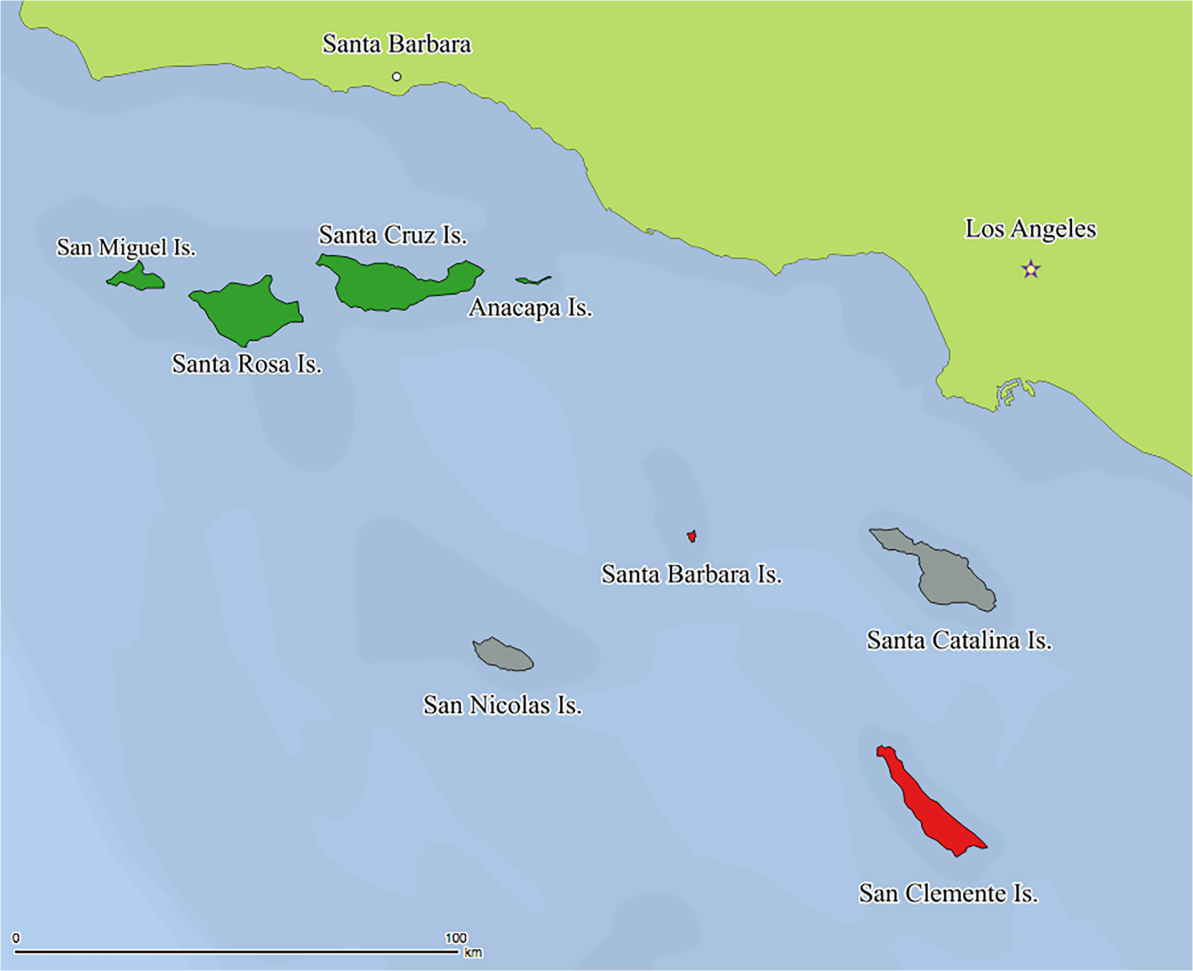
Map of the Californian Channel Islands showing the distribution of extant and extirpated breeding populations of Channel Island song sparrow. The archipelago includes the Northern Channel Islands: San Miguel, Santa Rosa, Santa Cruz and Anacapa Islands, and Southern Channel Islands: San Nicolas, Santa Barbara, Santa Catalina and San Clemente Islands. Song sparrows remain extant in the Northern Channel Islands (green) but were extirpated from Santa Barbara and San Clemente Islands (red). No breeding song sparrow populations have been reported on San Nicolas or Santa Catalina Islands (grey).

Isolation, novel ecological pressures and high degrees of endemism have made island populations important model systems for evolutionary and ecological studies [1]. In many taxa, adaptive and genetic divergence can occur over short geographic scales, such that near-shore islands can be of considerable conservation importance. The Californian Channel Island archipelago has been the focus of island ecologists and conservationists due to its high endemism and dynamic geological and disturbance history [2–4]. The Channel Islands are arranged as a northern and southern group the former as an east-west chain (Anacapa, Santa Cruz, Santa Rosa and San Miguel Islands), the latter is more isolated from the mainland and each other (Santa Catalina, Santa Barbara, San Clemente, San Nicolas; Fig 1). Sea-level changes during the Pleistocene and early Holocene altered the size and connectivity of the Channel Islands [3]. Up until 12 Kya, the northern group was joined as a single island called *Santarosae*, which was inundated into the five present day islands by rising sea-levels 9.6–9 Kya [2,3]. Although never connected, the water barrier separating the mainland and *Santarosae* has been as short as 7 km off-shore during the glacial maximum [3].

Including flora and fauna, there are approximately 140 endemic species or subspecies on the Channel Islands, that range from single-island endemics to endemics found across all islands [5–7]. Among the 56 breeding bird species on the Channel Islands there are 15 endemic avian subspecies and one endemic species, the island scrub jay (*Aphelocoma insularis*) [8].

Early hypotheses on patterns of colonization and subsequent divergence of the Channel Island avifauna were based on morphological divergence and biogeography [8]. Species with strong morphological divergence and restricted distributions such as the island scrub jay were predicted to have colonized the Channel Islands in a single event, followed by limited post-colonization gene flow [8,9]. Mitochondrial data from the island scrub jay confirmed genetic isolation from the mainland species, the western scrub jay (*Aphelicoma californica*), for more than 150 Kya [10]. Single endemic subspecies with multi-island distributions such as the endemic horned lark (*Eremophila alpestris insularis)* should have lower genetic divergence than taxa that had diverged into several endemic subspecies such as the loggerhead shrike (*Lanius ludovicianus anthonyi*). Genetic analyses from Channel Island populations of loggerhead shrikes [11,12] and horned larks [13] were consistent with these predictions.

Biogeographic patterns across the Channel Islands have been altered by significant disturbances ranging from widespread wildfires in the Pleistocene to extensive livestock overgrazing during the 19^th^ century [4,5,14,15]. During the 1960’s there were several avian extinctions: the San Clemente Island endemic subspecies of Bewick’s wren (*T*. *b*. *leucophyrus*) and song sparrow (*M*.*m clementae*), and the Santa Barbara Island song sparrow (*M*. *m*. *graminea)* [16,17]. The endemic loggerhead shrike subspecies *L*. *l*. *anthonyi* and *L*. *l*. *mearnsi* have been driven to precariously low population sizes with current estimates of less than 250 and 50 birds respectively [18].

This study focuses on the endemic Channel Island song sparrow which is currently recognized as a threatened, endemic subspecies *M*. *m*. *graminea* [16,19]. The Channel Island song sparrow populations were previously recognized as three subspecies: *M*.*m clementae* (Santa Rosa, San Clemente Islands), *M*.*m*. *micronyx* (San Miguel Island), and *M*. *m*. *graminea* (Santa Barbara Island); these subspecies were recognized on morphological characters [8]. Based on this taxonomy, morphological differences and distribution, it was predicted that song sparrows colonized the Channel Islands in a single event from the mainland with inter-island colonization but limited subsequent dispersal [8].

Throughout their range, song sparrows are highly polytypic with 25 diagnosable subspecies that range from c. 16–50g in body weight and display a wide variety of migratory and life history behaviors [20–22]. The geographic range of the different song sparrow subspecies varies from a single subspecies spanning thousands of kilometers [23] to five subspecies co-occurring within the San Francisco Bay [21]. However, song sparrow populations can become genetically divergent in the absence of morphological divergence over spatial scales of less than 10km [24]. The Channel Island song sparrow is non-migratory and based on the dispersal ecology of other non-migratory insular song sparrow populations, we predict low levels of contemporary dispersal between the extant populations of Channel Island song sparrows on Santa Rosa, San Miguel and Santa Cruz Islands.

We first use mitochondrial control region data to examine the patterns of genetic structure between extant (Santa Cruz, Santa Rosa and San Miguel Islands) and extirpated (Santa Barbara and San Clemente Islands) populations of the Channel Island song sparrow and several mainland sites. Secondly, we use microsatellite data from the extant populations to: determine contemporary inter-island patterns of gene flow, test for evidence of historic population bottlenecks and to compare the genetic structuring found in other mainland song sparrow populations.

## Materials and Methods

### Ethics statement

This work was approved by the University of British Columbia Animal Care Committee (Permit: A04-0177) and the Institutional Animal Care and Use Committee at the University of California—Santa Barbara (Permit 1-06-701). Fieldwork was conducted under permits from the United States Fish and Wildlife Service (Permit: 10596), California Department of Fish and Game (Permit: SC-008327) and United States National Park Service (Permit: CHIS-00049). Field protocols adhered to all welfare recommendations made by the Ornithological Council [25].

### Sample collection

We collected blood samples for genetic analysis from the extant song sparrow breeding populations on San Miguel, Santa Rosa and Santa Cruz Islands (Fig 1) and one mainland site at Coal Oil Point, Santa Barbara (see below) during the breeding season (March-April) of 2006 and 2007. Adults were captured using mist-nets with passive sampling or targeted playback. A 50 uL blood sample was collected from the brachial vein using a 31 gauge needle and a non-heparinized capillary tube. All individuals were banded with USFW bands and released once the brachial vein was no longer bleeding. No injuries or mortalities were noted during the field project. Contemporary sample sizes were: 20 for San Miguel (2006, 2007), 27 for Santa Rosa (2006), and 21 for Santa Cruz Islands (2006), and 10 for Coal Oil Point, Santa Barbara County (2007).

Toepad tissue from specimens collected from the extirpated populations on Santa Barbara (n = 11, 1938) and San Clemente Islands (n = 15, 1915) were obtained from the Museum of Vertebrate Zoology (UC Berkeley) and the San Diego Natural History Museum. The San Diego Natural History Museum also provided additional historic samples from the Californian (Los Angeles n = 1, 1928, San Diego n = 1, 1984, Ventura n = 3, 1920) and Mexican mainland (n = 3, 1923, 1958, 1997). Accession data is provided in S1 Table.

### Molecular methods

DNA was extracted from 78 contemporary blood samples using GenElute Blood Genomic DNA Miniprep Kit (Sigma–Aldrich). We extracted DNA from the museum toepads in a dedicated laboratory using QIAamp DNA micro kit (Qiagen). Contemporary samples were genotyped at nine microsatellites: Mme1, Mme2, Mme3, Mme7, Mme8, Mme12, Escu1, GF5 and PSAP335 [21]. Mme3 and Mme7 are z–linked loci; thus we scored the second allele as missing in the ten females. Microsatellite PCRs were performed in 15 μl volumes containing approximately 100 ng genomic DNA, 10 mm Tris–HCl (pH 8.3), 50 mm KCl, 1.5–2 mm MgCl2, 0.2 mm dNTPs (Invitrogen), 0.16 μg/μl bovine serum albumin, 0.1% Triton X–100 (Sigma–Aldrich), 1 pmol of each primer, 0.3 pmol of M13 Forward or Reverse IRDye 700 or 800 ((Li–Cor) and 0.5 U of Taq polymerase (Roche). PCR reactions were carried out using standard conditions and locus specific annealing temperatures (see Chan & Arcese 2002). Microsatellite PCR products were fractionated on 7% polyacrylamide gels using a Li–Cor 4200 DNA analyzer. Allele sizes were calibrated against a commercial size standard (50–350 bp, Li–Cor) and a species–specific allele ladder, and visualized using Base ImagIR (Li–Cor) and scored manually using RFLP scan (Scanalytics, CSP Inc.). Microsatellite data generated in this study is available at Figshare doi: dx.doi.org/10.6084/m9.figshare.1489740.

We obtained 500 bp of mtDNA sequence from a subset of the contemporary population samples and all of the extirpated population samples. Sample sizes for the sequence data were: Santa Cruz (n = 11), San Miguel (n = 10), Santa Rosa (n = 9), Santa Barbara (n = 11) San Clemente (n = 15) Islands, California mainland (n = 5) and the Mexican mainland (n = 3). All sequences are available in GenBank accession numbers: KT312851-312913.

In order to deal with the degraded DNA from museum specimens, we designed primers to amplify small (< 300 bp) overlapping segments of the mtDNA control region based on published song sparrow sequences [26]. Template amplifications were performed in 30 μl volumes containing 50 ng of DNA, 10 mm Tris–HCl (pH 8.3), 50 mm KCl, 2.5 mm MgCl_2_, 0.2 mm dNTPs (Invitrogen), 2.4 μg/μl BSA, 0.1% Triton X–100 (Sigma–Aldrich), 10 pmol of each primer and 1.0 U of Taq polymerase (Stratagene). The cycling profile was: 95°C for 1 min, 35 cycles of 95°C for 30 sec, 56°C for 30 sec, and 72°C for 45 sec, followed by a final extension for 5 min at 72°C. Sequencing reactions were performed commercially at Macrogen–Korea using Big Dye chemistry and an ABI3730 XL automatic DNA sequencer.

### Data analysis

Raw sequence data were edited, aligned and concatenated using Geneious v.8.0.5 [27] and collapsed to haplotypes using DnaSP v.5.10 [28]. Unique haplotypes were incorporated into a haplotype network using a median–joining minimum spanning network in PopART v1 [29]. To examine the genetic structure among the islands and the mainland populations we conducted an analysis of molecular variance (AMOVA) using Arlequin version 3.5 [30,31]. DnaSP v.5.10 [28] was used to calculate sequence-based genetic differentiation estimates (K_ST_) [32].

We tested for departures from Hardy–Weinberg equilibrium (HWE) and linkage equilibrium in the contemporary microsatellite data using Genepop v 3.4 [33], with tests run for each island, based on 100 batches and 1000 iterations. Null alleles were evaluated using MLNullFreq [34] and statistical tests corrected for multiple comparisons using the false discovery rate [35]. We calculated G''ST to estimate inter-island differentiation. G''ST is a standardized measure of Nei’s G’_ST_ [36], which corrects for within-population diversity and a small number of sampled populations [37]. Microsatellite data (from the same panel of markers) was available from our previous work for mainland *M*. *m*. *heermanni* populations sampled at the Salton Sea and San Francisco Bay [21,38], which augmented our single mainland site at Coal Oil Point, Santa Barbara Co., allowing us to estimate differentiation between extant Channel Island populations and three mainland sites. Differentiation calculations (G''_ST_) were performed using the GenAlex 6.5 software [39], and significance was based on 9,999 permutations.

We estimated migration rates among extant Channel Island populations using BayesAss v 2.3 [40]. We ran ten replicates of Markov Chain Monte Carlo (MCMC) chains 3×10^7^ iterations in length with a burn-in of 2×10^6^ iterations and collected estimates every 2,000 iterations to estimate the posterior distribution. We used delta values of 0.15, 0.15 and 0.20 for the migration rates, population allelic frequencies and inbreeding coefficient (F_IS_), respectively.

As a complement to BayesAss, we calculated population assignment probabilities using GeneClass2 [41]. The assignment probabilities were calculated based on the Bayesian criterion [42], and the Monte–Carlo re–sampling algorithm [43]. We also used the Bayesian clustering program Structure v. 2.3.3 [44] to estimate the number of genetically distinct clusters (K) among the three mainland sites (San Francisco Bay, Salton Sea and Coal Oil Point) and Santa Cruz, San Miguel and Santa Rosa Islands, assuming no–admixture and uninformative priors, and using 10 repetitions of 106 iterations with a burn–in of 10^5^ iterations for K = 1 to 6. The best K was inferred from posterior probabilities [44] by the ∆K method in Structure Harvester v.1 [45,46]. Clummp v. 1.2.2 [47] was used to summarize replicate runs, and a clustering graphic was created using Distruct v. 1.1 [48].

Given the dramatic habitat loss on the Channel Islands, we tested for the presence of genetic bottlenecks within the contemporary populations on San Miguel, Santa Rosa and Santa Cruz Islands using the program Bottleneck v. 1.2.02 [49,50]. We ran Bottleneck using the IAM mutation model, which best describes our loci (a mix of di–and imperfect dinucleotide repeats; [51]). Significance testing was based on 1000 iterations and the Wilcoxon signed rank test [52].

## Results

**Fig 2:**
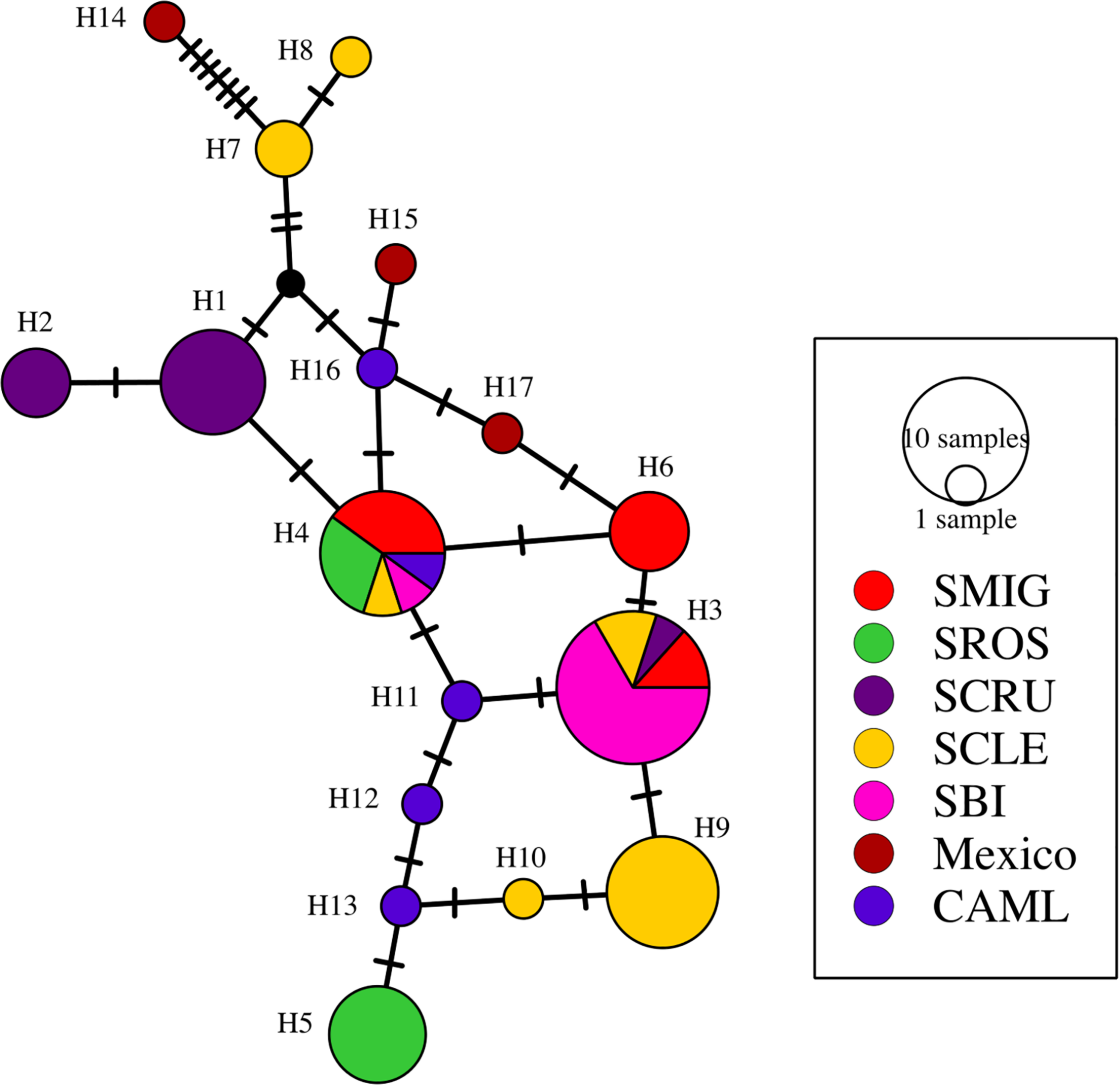
Median joining network of the mitochondrial control region haplotypes sampled from song sparrows in the Californian Channel Islands San Miguel Islands (SMIG), Santa Rosa (SROS), Santa Cruz (SCRU), San Clemente (SCLE), Santa Barbara (SBI) and mainland sites in California (CAML) and Mexico (Mexico). The haplotypes are colour–coded by sampling population, with size being proportionate to the number of individuals sampled with that haplotype. Each hatch mark or black circle between the haplotypes corresponds to a nucleotide substitution.

Ten mtDNA haplotypes were observed in 60 samples from Santa Cruz, Santa Rosa, San Miguel, San Clemente and Santa Barbara Islands. The eight samples from the Californian and Mexican mainland yielded seven additional unique haplotypes. Haplotypes 3 and 4 were shared across all islands in the northern and the southern island groups, and haplotype 4 was found in a specimen from Los Angeles (Fig 2). Haplotype 3 was sampled from Santa Cruz song sparrows in a previous study [26], but no other previously published haplotypes were detected within our Channel Islands samples.

Genetic divergence based on sequence differences revealed a closer relationship between the southern Channel Island populations on San Clemente and Santa Barbara, both to each other and to the mainland than to Santa Cruz and Santa Rosa populations (Table 1). The Santa Barbara population was more divergent from all populations than was the San Clemente population. The sequences found on Santa Rosa, Santa Cruz and San Miguel had a closer affinity to those found on the southern Channel Islands and mainland populations, than to each other, which led to these islands being more divergent from each other than from San Clemente and mainland populations.

**Table 1:** Pairwise genetic divergence (K_ST_) based on 500 bp of mitochondrial control region sequence from extant *M*. *m*. *graminea* populations on the northern Channel Islands (Santa Cruz, San Miguel and Santa Rosa), extinct *M*. *m*. *graminea* populations on the southern Channel Islands (San Clemente and Santa Barbara) and *M*. *m*. *heermanni* populations from sites on the Californian and Mexican mainland. Comparisons that were non-significant are highlighted.

	Californian ML	Mexican ML	Santa Cruz Is	Santa Rosa Is	San Miguel Is	San Clemente Is	Santa Barbara Is
Californian ML	0	** **				** **	
Mexican ML	**0.182**	0					
Santa Cruz Is	0.426	0.403	0				
Santa Rosa Is	0.215	0.282	0.498	0			
San Miguel Is	0.135	0.238	0.434	0.314	0		
San Clemente Is	**0.081**	0.163	0.371	0.287	0.236	0	
Santa Barbara Is	0.459	0.491	0.704	0.617	0.468	0.171	0

AMOVA analyses based on the mitochondrial data revealed that the majority of the genetic variation occurred among populations within island groups (F_SC_ = 0.32 p<0.001) and between populations (F_ST_ = 0.48 p<0.001) and not between the northern and southern island groups (F_CT_ = 0.24 p = 0.09).

Microsatellite data from the extant populations were in HWE and linkage equilibrium after correcting for false discovery rates [35]. No null alleles were detected. Pairwise differentiation (G''_ST_) was statistically significant for all inter-island comparisons (Table 2). Differentiation between Santa Rosa and Santa Cruz populations was modest (G''_ST_: 0.14), but Santa Rosa and San Miguel populations were more diverged (G''_ST_: 0.37), and San Miguel and Santa Cruz were highly diverged (G''_ST_: 0.45). San Miguel sparrows were also the most diverged from mainland populations (G''_ST_ = 0.64, 0.52, 0.43 for San Francisco Bay, Salton Sea and Coal Oil Point respectively; Table 2). Santa Cruz and Santa Rosa were similarly but slightly less diverged from San Francisco Bay, Salton Sea and Coal Oil Point populations, respectively (Table 2).

**Table 2:** Pairwise genetic divergence (G''_ST_) based on nine microsatellite loci from extant *M*. *m*. *graminea* populations on the Northern Channel Islands (Santa Cruz, San Miguel and Santa Rosa Islands) and three mainland populations (*M*. *m*. *heermanni*) from San Francisco Bay, Salton Sea and Coal Oil Point, Santa Barbara Co. All comparisons were significant after correction for false discovery rate (P < 0.04).

	San Francisco Bay	Salton Sea	Santa Barbara Co.	Santa Cruz Is	San Miguel Is	Santa Rosa Is
*M*.*m*.*heermanni*						
San Francisco Bay	0					
Salton Sea	0.12	0				
Santa Barbara Co.	0.27	0.18	0			
*M*.*m*.*graminea*						
Santa Cruz Is	0.43	0.33	0.30	0		
San Miguel Is	0.64	0.52	0.43	0.45	0	
Santa Rosa Is	0.40	0.32	0.34	0.14	0.37	0

Estimated migration rates into San Miguel and Santa Rosa Island populations from all other donor populations were less than 0.011 (BayesAss, Table 3). Santa Cruz Island appears to receive higher immigration from Santa Rosa (m_C_ = 0.3), a result supported by GeneClass2 assignment tests. In the GeneClass2 analysis for detection of first–generation migrants, one individual from Santa Cruz was assigned to Santa Rosa (p = 0.001) and another from Santa Rosa was assigned to Santa Cruz (p = 0.002), while all other individuals were assigned to the island from where they were sampled.

The Bottleneck analyses were statistically significant on San Miguel, Santa Rosa, Santa Cruz Islands (p = 0.013, 0.013, 0.037; respectively), which supports the occurrence of historic bottlenecks in each population.

## Discussion

### Genetic divergence patterns between extant and extirpated populations

The Channel Island song sparrow is a species of special concern in California [16], and this study provides genetic data from both extant and extirpated populations to examine past hypotheses on island colonization and provides important information on the current population genetic structure and inferred dispersal patterns. Our mitochondrial data from extant (Santa Cruz, Santa Rosa and San Miguel Islands), extirpated (Santa Barbara and San Clemente) and mainland populations provided additional support for the genetic uniqueness of the Channel Island populations. However, additional mainland sampling revealed that the islands do share haplotypes with the mainland populations of *M*. *m*. *heermanni* and are not reciprocally monophyletic. The haplotype network and pattern of genetic divergence between northern and southern Channel Island and mainland populations are consistent with various scenarios of asymmetrical inter-island migration with subsequently low dispersal. Our data, however, could also be consistent with separate mainland colonizations to the northern and southern groups or repeated immigration from the same unsampled mainland source. The phylogenetic signal has likely been affected by drift, given the low diversity found on the smaller islands and the presence of divergent haplotypes on neighbouring islands (Table 1, Fig 2).

Our results are similar to the mitochondrial haplotype data from other Channel Island endemic subspecies such as horned larks and loggerhead shrikes that also shared haplotypes between the northern and southern Channel Islands and mainland populations [11–13]. The microsatellite results from loggerhead shrikes are comparable to song sparrows [12]; both species displayed considerable genetic divergence from the mainland population, but direct comparison is limited by differences in marker variability [37] and sampling design.

Compared to other song sparrow insular subspecies, the Channel Island song sparrow populations demonstrate morphological and genetic divergence over comparatively small water barriers. This contrasts with the coastal archipelagos of Alaska and British Columbia, that show morphological and genetic divergence over much larger geographic scales [23,24]. Within California, genetic divergence between the adaptively different coastal subspecies (*M*. *m*. *heermanni*) and the salt marsh specialist (*M*. *m*. *pusillula*), was much lower (G''_ST_: 0.22–0.25) than the divergence between *M*. *m*. *heermanni* and the Channel Island subspecies *M*. *m*. *graminea* (G''_ST_: 0.40–0.64) studied here (see also [21,53]).

Fossil evidence indicates song sparrows were on the islands as long as 25–39 Kya [54], so a combination of long periods of reduced gene flow and small population sizes would increase the drift-mediated genetic divergence of the Channel Island song sparrows. Our data does not enable us to examine adaptive divergence, but other Channel Island taxa have shown rapid morphological divergence with adaptive significance [55,56].

### Contemporary inter-island dispersal

Genetic estimates of immigration can have substantial relevance to conservation biology by estimating the importance of immigration rates to the persistence of particular populations [57]. Despite haplotype sharing between the northern and southern group, the extent of genetic structure among the northern islands and the absence of any recolonization to Santa Barbara or San Clemente Islands indicates that inter-island and mainland immigration is limited over the time scale relevant for population management.

The pattern of increasing genetic divergence and decreased migration (this study) and allelic diversity along the northern Channel Islands [58] is consistent with a model of stepping stone migration with limited subsequent gene flow from mainland sources. As the terminus in the chain, San Miguel Island has low genetic diversity, negligible unique alleles [58], substantial structuring and low estimated migration rates. Our results suggest that the San Miguel Island song sparrow population received limited gene flow from other islands and is likely demographically independent. The mitochondrial data from this study also supports the presence of low gene flow and drift effects across the northern group, for example, Santa Rosa and Santa Cruz are geographically close, but were more genetically divergent to each other than to populations in the southern group.

Although water barriers are known to limit song sparrow dispersal, other factors such as the dominant northwesterly pattern of wind in the region [9], biogeography [59] or habitat structure may alter dispersal and colonization patterns. For example, prior to 1950, song sparrows were rare or absent on Santa Cruz Island, potentially due to competitive exclusion by rufous–crowned sparrows (*Aimophila ruficeps* [59]). However, song sparrows now breed on Santa Cruz, perhaps due to changing habitat structure resulting from management activities that have also affected other Channel Island avifauna [60].

**Fig 3:**
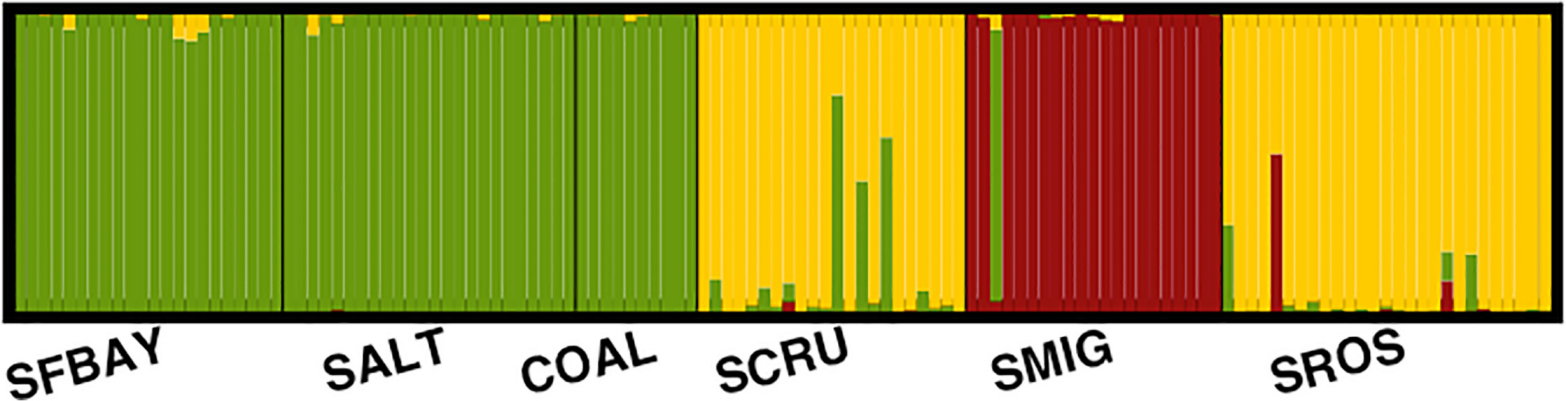
Bayesian clustering analysis of *M*. *m*. *heermanni* populations in San Francisco Bay (SFBAY), Salton Sea (SALT) and Santa Barbara (COAL) and *M*. *m*. *graminea* populations on Santa Cruz (SCRU), Santa Rosa (SROS) and San Miguel Islands (SMIG). The proportions of the bars indicate the proportion of ancestry for each individual that is attributed each of the three clusters.

Estimated migration rates (Table 3) and Bayesian clustering (Fig 3) indicate that the Santa Rosa population was either the source, or augmented the song sparrow population on Santa Cruz Island. Some contribution of mainland gene flow to the Santa Cruz population is also supported by the cluster analysis (Fig 3). Interestingly, the Santa Cruz song sparrow specimens have been described as being morphologically intermediate between the Santa Rosa and mainland populations [61].

**Table 3:** Estimated contemporary migration (m_C_; BayesAss) among Channel Island song sparrow populations. The mode of migration rate is provided with its 95% credible interval.

Recipient population	Donor population	m_C_ (95% CI)
San Miguel	San Miguel	0.982 (0.93–1.00)
	Santa Rosa	0.011 (0.00–0.049)
	Santa Cruz	0.008 (0.00–0.034)
Santa Rosa	San Miguel	0.006 (0.00–0.028)
	Santa Rosa	0.987 (0.96–1.00)
	Santa Cruz	0.007 (0.00–0.031)
Santa Cruz	San Miguel	0.014 (0.00–0.048)
	Santa Rosa	0.301 (0.25–0.33)
	Santa Cruz	0.685 (0.67–0.73)

Analyses using Structure suggested three genetic clusters (Fig 3; K = 3; consensus of 10 replicates). Santa Cruz and Santa Rosa Island populations exhibited almost complete membership in the same cluster, whereas 19 birds from San Miguel were all assigned to a second cluster. Three birds from Santa Cruz Island and a single bird from San Miguel had a high membership in the mainland cluster, which supports some previous mainland migration. The available mainland samples from San Francisco Bay, Salton Sea and Coal Oil Point formed a third distinct genetic cluster.

Population bottlenecks also contribute to high genetic divergence [62]; for example, the San Clemente loggerhead shrike declined to ~ 15 birds in 1999 and their contemporary population genetic structure reflects this [12]. In this study, we tested for, and found genetic bottlenecks within the Santa Rosa, Santa Cruz and San Miguel Island populations. In the 19^th^ century, extensive livestock grazing and invasive species introduction had substantial impacts on many Channel Island species, so detecting genetic bottlenecks in the song sparrow populations was expected. Although the late Pleistocene wildfires and inundation of *Santarosae* would have impacted song sparrow population sizes, the genetic bottleneck signal is detectable for 2-4N_e_ generations after the bottleneck, so our analyses likely reflect more recent history [52].

Non-migratory song sparrows often show high site fidelity and limited natal dispersal [63–65]; therefore, the patterns of genetic divergence of the Channel Island song sparrow presented in this study provide a baseline for the degree to which immigration might contribute to population stability and the potential for genetic divergence in other Channel Island avifauna. The concurrent estimation of neutral and adaptive genetic divergence has great potential to facilitate conservation management in the Channel Islands, but also to unravel the complex microevolutionary histories of the endemic taxa that may be obscured by population turnover and genetic drift [66].

## Supporting Information

S1 Table Museum sampling information with corresponding haplotype designation. (DOCX)
