# Supplementary material for: Genetic Divergence of an Avian Endemic on the Californian Channel Islands
Source: PLoS One. 2015 Aug 26;10(8):e0134471. doi: 10.1371/journal.pone.0134471 (PMC4550415; doi:10.1371/journal.pone.0134471)
Supplement: S1 Table — (DOCX) [file pone.0134471.s001.docx]

**Table S1. Specimen voucher information from the San Diego Museum of Natural History (SDMNH) and the University of California at Berkeley’s Museum of Vertebrate Zoology (MVZ)**

| Specimen Voucher | Collection Date | Location |
| --- | --- | --- |
| SDMNH:35020 | 23-03-1915 | San Clemente Is. |
| SDMNH:1125 | 25-08-1894 | San Clemente Is. |
| SDMNH:35025 | 29-03-1915 | San Clemente Is. |
| SDMNH:1123 | 22-08-1894 | San Clemente Is. |
| SDMNH:29714 | 25-08-1894 | San Clemente Is. |
| SDMNH:35021 | 27-03-1915 | San Clemente Is. |
| SDMNH:106618 | 05-04-1914 | San Clemente Is. |
| SDMNH:35024 | 29-03-1915 | San Clemente Is. |
| SDMNH:108147 | 29-08-1908 | San Clemente Is. |
| SDMNH:10197 | 14-12-1925 | San Clemente Is. |
| SDMNH:83197 | 28-03-1915 | San Clemente Is. |
| SDMNH:36073 | 11-04-1897 | San Clemente Is. |
| SDMNH:106619 | 30-04-1914 | San Clemente Is. |
| SDMNH:35026 | 31-03-1931 | San Clemente Is. |
| SDMNH:10189 | 13-12-1925 | San Clemente Is. |
| MVZ:81594 | 15-05-1927 | Santa Barbara Is. |
| MVZ:17873 | 19-04-1938 | Santa Barbara Is. |
| MVZ:29712 | 06-04-1906 | Santa Barbara Is. |
| MVZ:106615 | 29-05-1914 | Santa Barbara Is. |
| MVZ:76171 | 15-04-1939 | Santa Barbara Is. |
| MVZ:17868 | 19-04-1938 | Santa Barbara Is. |
| MVZ:56232 | 10-08-1930 | Santa Barbara Is. |
| MVZ:81595 | 15-05-1927 | Santa Barbara Is. |
| MVZ:81593 | 16-05-1927 | Santa Barbara Is. |
| MVZ:29713 | 08-03-1909 | Santa Barbara Is. |
| MVZ:81592 | 15-05-1927 | Santa Barbara Is. |
| SDMNH:29724 | 20-05-1928 | Culver City, Los Angeles Co |
| SDMNH:34950 | 26-02-1920 | Ventura |
| SDMNH:34954 | 26-02-1920 | Ventura |
| SDMNH:34955 | 26-02-1920 | Ventura |
| SDMNH:42928 | 25-02-1984 | San Luis Rey River, San Diego Co. |
| SDMNH:8723 | 06-05-1923 | Las Cabras, Baja Mexico |
| SDMNH:30077 | 01-03-1958 | Mexico: San Fernando Mission, Mexico |
| SDMNH:51106 | 03-10-1997 | San Jose de Magdelena, Mexico |
